# FDG-PET parameters predict for recurrence in anal cancer – results from a prospective, multicentre clinical trial

**DOI:** 10.1186/s13014-019-1342-9

**Published:** 2019-08-06

**Authors:** Michael Peter Jones, George Hruby, Ur Metser, Swetha Sridharan, Anne Capp, Mahesh Kumar, Sarah Gallagher, Natalie Rutherford, Carl Holder, Christopher Oldmeadow, Jarad Martin

**Affiliations:** 1 WP Holman Clinic, Royal Hobart Hospital, Hobart, Tasmania Australia; 2 The University of Newcastle, Callaghan, New South Wales Australia; 3 Department of Radiation Oncology, Royal North Shore Hospital, St Leonards, New South Wales Australia; 4 Department of Medical Imaging, Princess Margaret Cancer Centre, Toronto, Ontario Canada; 5 Department of Radiation Oncology, Calvary Mater Newcastle, Waratah, New South Wales Australia; 6 Department of Nuclear Medicine, Calvary Mater Newcastle, Waratah, New South Wales Australia; 7 Hunter Medical Research Institute, New Lambton Heights, New South Wales Australia

**Keywords:** Anal cancer, Positron emission tomography, Chemo-radiotherapy

## Abstract

**Background:**

To investigate the prognostic significance of positron emission tomography (PET) parameters from F-18 fluorodeoxyglucose (FDG) PET scans performed pre- and post- chemo-radiotherapy (CRT) for squamous cell carcinoma of the anal canal (AC).

**Methods:**

From January 2013 to January 2017, 19 patients with non-metastatic AC enrolled on a prospective trial underwent FDG-PET/CT imaging before and 12 weeks following CRT. A computer-generated volume of interest (VOI) was snapped around the primary tumour using six different standard uptake value (SUV) thresholds and the following parameters were extracted: SUV max, mean, median, standard deviation and peak as well as metabolic tumour volume (MTV) and total lesion glycolysis. Exact logistic regression and ROC AUC analyses were performed for each metric at each timepoint.

**Results:**

With a median follow up of 15.8 months, 3/19 patients had a local recurrence and 5/19 had any recurrence. On post-CRT PET, the median SUV within a VOI bounded by an SUV of 3 correlated with local recurrence (*p* < 0.01) and demonstrated excellent discrimination (ROC AUC 1.00, perfect separation was achieved at a median SUV of 3.38). The mean SUV at this threshold did not quite reach significance for prediction of local recurrence (*p* = 0.06) but demonstrated excellent discrimination (ROC AUC 0.91). The MTV bounded by a threshold of 41% SUVmax on the pre-CRT PET predicted for any recurrence (*p* = 0.03) and showed excellent discrimination (ROC AUC 0.89).

**Conclusions:**

FDG-PET parameters are predictive of recurrence in AC. FDG-PET may represent a valuable tool for prognostication and response assessment in AC.

**Trial registration:**

ANZCTR, ACTRN12614001219673. Registered 19 November 2014 - Retrospectively registered.

## Background

Squamous cell carcinoma of the anal canal (AC) is an uncommon tumour with increasing incidence [1]. Standard treatment is definitive chemo-radiotherapy which allows for organ preservation [2, 3], with abdomino-perineal resection (APR) reserved for salvage.

Fluorodeoxyglucose-positron emission tomography/computed tomography (FDG-PET/CT) is recommended for both staging and radiotherapy planning in AC [4]. It is also being used by some groups for response assessment. However, current evidence to support this practice is principally limited to retrospective studies using visual PET analysis of scans performed at widely variable time points [5–9]. As such, the role of post-CRT FDG-PET/CT imaging remains controversial with questions remaining over scan timing and their interpretation.

Quantitative analysis of PET parameters represents a more accurate, objective, and reproducible means of assessing PET images. Retrospective studies in AC patients extracting PET parameters from pre-CRT PET imaging have correlated maximum standardized uptake value (SUV) and metabolic tumour volume (MTV) with patient outcomes [10–14]. Similar studies have not yet been performed on post-CRT PET imaging.

Clinical examination remains the cornerstone of local response assessment following definitive CRT [2]. Close regular follow-up is critical as patients who develop a local recurrence may be potential candidates for salvage APR. Thus, an imaging biomarker could help identify patients at increased risk of recurrence and allow for personalisation of follow-up schedules. We sought to determine whether PET parameters on pre- and/or post-CRT PET scans could predict for recurrence in a cohort of AC patients treated uniformly in a multi-centre prospective clinical trial.

## Methods

### Patients

Patients with histologically confirmed non-metastatic squamous cell carcinoma of the anal canal were enrolled into a prospective study at three Australian centres investigating the role of multi-parametric magnetic resonance imaging (MRI) as a biomarker in AC. The protocol has been previously published [15]. Patients were staged according to the AJCC Cancer Staging Manual, Seventh Edition. p16 testing was performed on all tumours. Informed consent was obtained from all participants. The study was approved by the Hunter New England Human Research Ethics Committee (reference: HREC/12/HNE/408) and prospectively registered (ACTRN12614001219673).

### Chemo-radiotherapy

Patients were treated with Intensity Modulated Radiotherapy (IMRT) or Volumetric Modulated Arc Therapy (VMAT). Prescription dose, contouring, and radiotherapy planning were mandated as per the Australasian Gastrointestinal Trials Group (AGITG) guidelines [16]. Radiotherapy doses ranged from 50.4-54Gy, depending on tumour stage. Continuous infusional 5-Fluorouracil (5-FU) was delivered during the first and fourth weeks of radiation treatment at a dose of 800-1000 mg/m^2^/day over 5 days. Mitomycin-C at 10 mg/kg was delivered on day one only.

### FDG-PET/CT imaging and analysis

FDG-PET/CT was performed at the time of staging (pre-CRT) and 12 weeks post-CRT. Images were acquired on a GE 690, GE 710 or Siemens Biograph mCT PET/CT. Patients were fasted for a minimum of 6 h prior to scanning to ensure a blood glucose level of < 10 mmol/L. Scanning was performed 1 h following FDG injection and extended from vertex of scalp to mid-thigh.

PET images were analysed utilising Mirada Medical workstation [Mirada Medical Ltd., Oxford, UK; software version Mirada XD3 (64-bit)]. A computer-generated volume of interest (VOI) was snapped around the primary tumour only (not involved lymph nodes) using six SUV thresholds from which seven PET parameters were extracted (Table 1).

**Table 1:** FDG-PET/CT parameters extracted using absolute and relative SUV thresholds

SUV Parameter	SUV Threshold
Maximum	3
Mean	5
Median	10
Standard deviation	41% maximum
Peak	65% maximum
Metabolic tumour volume	80% maximum
Total lesion glycolysis	

*SUV* Standard uptake value

### Statistics

Exact logistic regression models were used to assess the ability of the PET parameters to predict local and any recurrence; *p*-values and Receiver Operator Characteristic Area Under the Curve (ROC AUC) values are reported. A ROC AUC between 0.6–0.7, 0.7–0.8, 0.8–0.9, and 0.9–1.0 was considered to have poor, fair, good, and excellent discrimination performance, respectively. We also performed an elastic net LASSO logistic regression at each time point for all the PET parameters to determine if there was any combination of parameters that were predictive of recurrence. LASSO estimation is a method of constraining the parameter estimates such that they are less prone to over-fitting when the number of parameters is large relative to the number of events; variables that have no predictive ability have coefficients that are “shrunk” all the way to zero.

## Results

### Patients

Between January 2013 and January 2017, 19 patients were enrolled on the prospective study and underwent FDG-PET/CT imaging pre- and post- CRT. Patient and tumour characteristics are displayed in Table 2. All tumours were p16 positive.

**Table 2:** Patient characteristics

	No.	%
Gender		
Male	3	15.8
Female	16	84.2
Age		
Median	58.5	
Range	38–77	
Clinical T stage		
T1	1	5.3
T2	9	47.4
T3	5	26.3
T4	4	21.1
Clinical N stage		
N0	5	26.3
N1	6	31.6
N2	7	36.8
N3	1	5.3

### Outcomes

Medium follow-up was 15.8 months (range: 8.2–54.3). Three patients (15.8%) developed a local recurrence, one of whom also experienced regional and distant recurrence. Two patients developed distant metastatic disease without local recurrence for a total of five patients (26.3%) with any recurrence. At last follow-up, one patient had died of disease.

### FDG-PET parameters

**Fig. 1:**
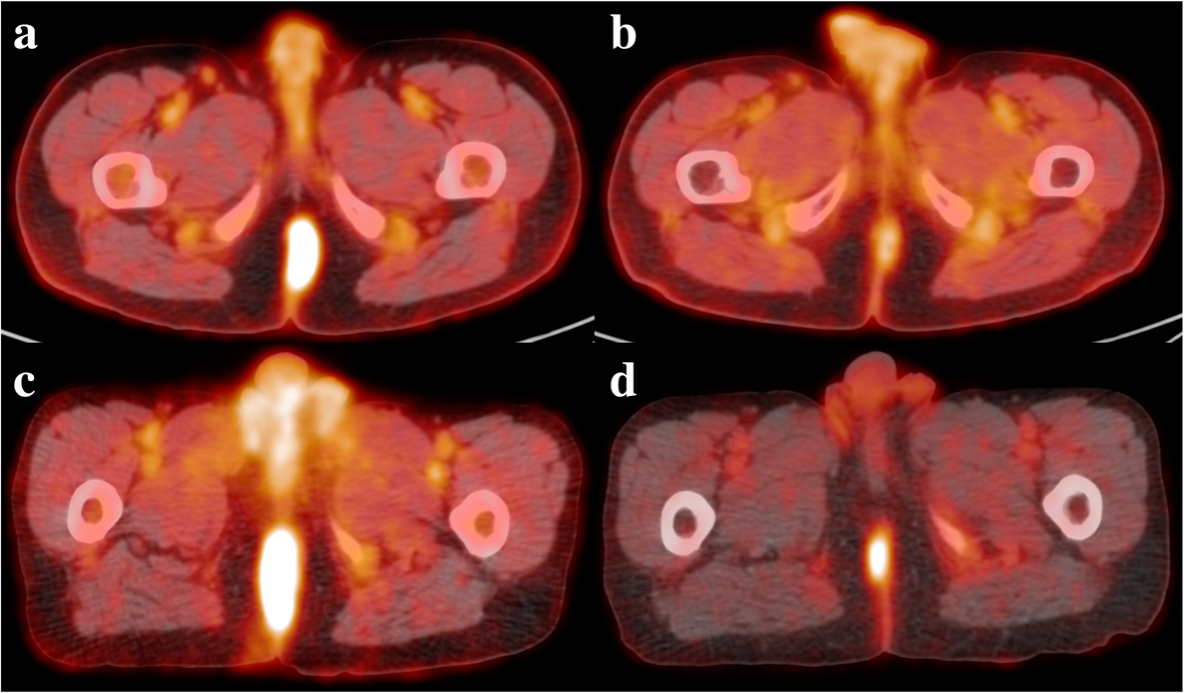
Pre- (**a**) and post- (**b**) chemo-radiotherapy FDG-PET/CT of a patient who did not experience a local recurrence. Pre- (**c**) and post- (**d**) chemo-radiotherapy FDG-PET/CT of a patient who subsequently experienced a local recurrence

Post-CRT PET parameters extracted from a VOI bounded by an SUV of 3 demonstrated the most promise (Table 3). The median SUV was the only statistically significant parameter for local recurrence (*p* < 0.01) and showed excellent discrimination (ROC AUC 1.00). All patients who had a median SUV greater than 3.4 experienced a local relapse. Median SUV did not reach statistical significance for detection of any recurrence (*p* = 0.07) and showed only fair discrimination (ROC AUC 0.75). The mean SUV at this threshold did not quite reach significance for prediction of local recurrence (*p* = 0.06) but displayed excellent discrimination (ROC AUC 0.91). For prediction of any recurrence, the only parameter reaching statistical significance was the MTV bounded by 41% maximum SUV on the pre-CRT PET (*p* = 0.03) which also showed excellent discrimination (ROC AUC 0.89). None of the pre-CRT PET scan parameters correlated with local recurrence. On LASSO regression, the only parameter retained was the SUVmax on the post-CRT PET. Figure 1 shows example FDG-PET/CT imaging in a patient who did (a-b), and a patient who did not (c-d), subsequently experience a local recurrence.

**Table 3:** Post-CRT FDG-PET/CT results for local recurrence using an SUV threshold of 3

SUV Parameter	*P*-value	AUC
Max	0.26	0.76
Mean	0.06	0.91
Median	< 0.01	1.00
Standard deviation	0.32	0.73
Peak	0.38	0.67
Volume	0.17	0.66
Total lesion glycolysis	0.16	0.67

*CRT* Chemo-radiotherapy, *SUV* Standard uptake value, *AUC* Area under the curve

## Discussion

Herewith we report the utility of both pre- and post-CRT PET parameters for outcome prediction in a cohort of AC patients. The patients were uniformly treated according to mandated contouring and dosing guidelines on a prospective multi-centre trial with FDG-PET/CT scans performed at the time of staging, and at 12 weeks post-completion of CRT.

Due to the small sample size, no definitive conclusions can be made regarding any particular parameter, but our findings provide several valuable insights. Firstly, post-CRT PET imaging appears to better predict for local recurrence than does pre-CRT imaging. Secondly, while a volumetric parameter (MTV) was more predictive of recurrence on pre-CRT PET imaging (in keeping with some previous studies), SUV parameters were more predictive on post-CRT imaging. Thirdly, PET parameters extracted from the primary tumour of post-CRT images better predict local than any recurrence, as may be expected, however this was not true for the pre-CRT images.

### Quantitative PET imaging

SUVmax is the most commonly used PET parameter in clinical practice. It is simple and quick to measure but is subject to noise because its value is taken from a single voxel. Alternatively, SUV mean, median or standard deviation within a VOI bounded by a threshold SUV may be more robust [17]. SUV thresholds for bounding VOI may be absolute (e.g. 3, 5, or 10) or relative to the SUVmax of the tumour. Another PET parameter in use is the peak SUV which is the average SUV contained within a small VOI approximating 1 cm^3^ that encompasses the voxels surrounding the SUVmax.

Volumetric PET parameters include the MTV, a measure of metabolic tumour volume above a threshold SUV, and total lesion glycolysis (TLG), which is the product of the mean SUV and MTV. Many feel that MTV and TLG better reflect metabolic tumour burden [17] and studies that have correlated MTV and TLG with prognosis [18–20] have included both cervical and head and neck cancer treated with CRT [21, 22].

### Pre-treatment PET imaging in anal cancer

FDG-PET/CT is recommended for AC staging and radiotherapy planning [2, 4]. Retrospective studies have correlated staging PET parameters with outcome in AC. Kidd et al. reported that a higher SUVmax was associated with an increased risk of lymph node metastases and worse disease-free survival [10]. In a smaller study, Deantonio et al. also showed that higher SUVmax predicted for more advanced stage but were unable to demonstrate any correlation with survival [11].

Three other studies were unable to correlate SUVmax with outcome [12–14]. They did however correlate MTV with outcome, although at different cut-off values. This is likely due to varying techniques used to calculate the MTV. Bazan et al. used a threshold of 50% SUVmax, included the primary and any nodal disease, and found a cut-point of 26 ml correlated with PFS [12]. Gauthe and colleagues also used a threshold of 50% SUVmax but only included the primary tumour. They found a cut-off of 7 ml correlated with overall survival [13]. Shali et al. included all metabolically active disease (primary, lymph nodes, and distant metastases) and used a threshold to define the volume of 30% of the SUVmax [14]. Not surprisingly, the MTV cut-off was higher (45 ml) than the others.

While these studies suffered from a retrospective design and heterogenous treatments, we too found that a volumetric parameter on the pre-treatment PET better correlated with recurrence than an SUV parameter. Our volume was bounded by 41% of the SUVmax and surrounded the primary tumour only. We chose to focus on the primary tumour as this is the most common site of recurrence [23–25] and is more practical to measure in routine clinical practice. We also pre-determined which parameters to examine prior to performing our analysis in order to reduce the impact of multiple statistical testing.

### Post-treatment PET imaging in anal cancer

Retrospective studies have investigated the predictive value of post-treatment PET scans in AC [5–9]. While most have reported that a complete response on PET correlated with improved survival, all suffered from heterogeneous treatment, variable timing of PET imaging, and visual assessment only. Following definitive treatment, the timing of PET imaging in particular can have a significant impact on the sensitivity and specificity.

This was highlighted in a thorough prospective study by Mistrangelo et al. who performed PET imaging and anal biopsies at 1- and 3-months post completion of CRT on 53 patients [26]. Again, treatment techniques varied and imaging assessment was qualitative, but compared to 1 month, PET imaging at 3 months was found to have both improved sensitivity (66.6 vs. 100%) and specificity (92.5 vs. 97.4%) for detection of persistent disease.

The timing of post treatment PET imaging in the published retrospective studies ranged from 0.9–5.4 months [5], 2-7 months [6], 1-8 months [7], 1-8 months [8], and 1-6 months [9]. This significantly undermines the ability to draw robust conclusions from their data and limits its application to clinical practice.

Cardenas et al. is the only published study to perform quantitative analysis of post-treatment PET imaging [27]. Treatment was non-standardised, the only parameter assessed was SUVmax and, unfortunately, they fail to report a range for the timing of PET imaging. In addition, their follow-up ranged from 3.6–94.1 months, which is less than the median time to PET imaging (3.8 months). As such, our analysis is both novel and lacks some of the weaknesses present in the current literature.

While our study has many strengths, its major limitation is the small sample size which reflects the scarcity of AC. We also acknowledge that extracting seven PET parameters at six SUV thresholds generated a large amount of data which increased the likelihood of finding significant outcomes. However, such an exploratory analysis is useful for identifying signals which warrant further investigation in larger patient cohorts, which was the aim of this preliminary study.

That FDG-PET/CT imaging was performed on three different PET/CT systems is both a strength and weakness. While it introduces more variables, our results are likely more robust, they reflect real world practice, and are hence more applicable in the community. Future studies could consider including all metabolically active disease (primary and nodes) which may (or may not) better predict for any recurrence.

Finally, the emergence of digital PET/CT with enhanced image quality, greater sensitivity, increased signal to noise ratio, faster time-of-flight resolution, and improved quantitative accuracy means that PET/CT will represent an increasingly attractive biomarker in the future [28].

## Conclusions

PET-parameters extracted from the primary tumour on FDG-PET/CT scans performed pre- and at 12 weeks following completion of CRT for AC correlate with recurrence and warrant further investigation in larger patient cohorts.

## Data Availability

The datasets analysed during the current study are available from the corresponding author on reasonable request.

## Abbreviations

AC: Anal cancer
AGITG: Australasian Gastrointestinal Trials Group
APR: Abdomino-perineal resection
AUC: Area under the curve
CRT: Chemo-radiotherapy
CT: Computed tomography
FDG: Fluorodeoxyglucose
IMRT: Intensity modulated radiation therapy
MRI: Magnetic resonance imaging
MTV: Metabolic tumour volume
PET: Positron emission tomography
ROC: Receiver operating characteristics
SUV: Standardised uptake value
TLG: Total lesion glycolysis
VMAT: Volumetric modulated radiation therapy
VOI: Volume of interest

## References

[1] Jemal A, Simard EP, Dorell C, Noone A-M, Markowitz LE, Kohler B (2013). Annual report to the nation on the status of cancer, 1975-2009, featuring the burden and trends in human papillomavirus(HPV)-associated cancers and HPV vaccination coverage levels. J Natl Cancer Inst.

[2] National Comprehensive Cancer Network (2018). Anal carcinoma (version 2.2018).

[3] Glynne-Jones R, Nilsson PJ, Aschele C, Goh V, Peiffert D, Cervantes A (2014). Anal cancer: ESMO–ESSO–ESTRO clinical practice guidelines for diagnosis, treatment and follow-up. Radiother Oncol.

[4] Jones M, Hruby G, Solomon M, Rutherford N, Martin J (2015). The role of FDG-PET in the initial staging and response assessment of anal cancer: a systematic review and meta-analysis. Ann Surg Oncol.

[5] Schwarz JK, Siegel BA, Dehdashti F, Myerson RJ, Fleshman JW, Grigsby PW (2008). Tumor response and survival predicted by post-therapy FDG-PET/CT in anal cancer. Int J Radiat Oncol Biol Phys.

[6] Nguyen BT, Joon DL, Khoo V, Quong G, Chao M, Wada M (2008). Assessing the impact of FDG-PET in the management of anal cancer. Radiother Oncol.

[7] Day FL, Link E, Ngan S, Leong T, Moodie K, Lynch C (2011). FDG-PET metabolic response predicts outcomes in anal cancer managed with chemoradiotherapy. Nat Publ Group.

[8] Houard C, Pinaquy J-B, Mesguich C, Henriques de Figueiredo B, Cazeau A-L, Allard J-B (2017). Role of 18F-FDG PET/CT in posttreatment evaluation of anal carcinoma. J Nucl Med.

[9] Goldman KE, White EC, Rao AR, Kaptein JS, Lien WW (2016). Posttreatment FDG-PET-CT response is predictive of tumor progression and survival in anal carcinoma. Pract Radiat Oncol.

[10] Kidd EA, Dehdashti F, Siegel BA, Grigsby PW (2010). Anal cancer maximum F-18 fluorodeoxyglucose uptake on positron emission tomography is correlated with prognosis. Radiother Oncol.

[11] Deantonio L, Milia ME, Cena T, Sacchetti G, Perotti C, Brambilla M (2015). Anal cancer FDG-PET standard uptake value: correlation with tumor characteristics, treatment response and survival. La radiologia medica.

[12] Bazan JG, Koong AC, Kapp DS, Quon A, Graves EE, Loo BW (2013). Metabolic tumor volume predicts disease progression and survival in patients with squamous cell carcinoma of the anal canal. J Nucl Med.

[13] Gauthé M, Richard-Molard M, Fayard J, Alberini J-L, Cacheux W, Lièvre A (2016). Prognostic impact of tumour burden assessed by metabolic tumour volume on FDG PET/CT in anal canal cancer. Eur J Nucl Med Mol Imaging.

[14] Shali SM, Schmitt V, Behrendt FF, Winz OH, Heinzel A, Mottaghy FM (2016). Metabolic tumour volume of anal carcinoma on 18FDG PET/CT before combined radiochemotherapy is the only independant determinant of recurrence free survival. Eur J Radiol.

[15] Jones M, Hruby G, Stanwell P, Gallagher S, Wong K, Arm J (2015). Multiparametric MRI as an outcome predictor for anal canal cancer managed with chemoradiotherapy. BMC Cancer.

[16] Ng M, Leong T, Chander S, Chu J, Kneebone A, Carroll S (2012). Australasian gastrointestinal trials group (AGITG) contouring atlas and planning guidelines for intensity-modulated radiotherapy in anal cancer. Int J Radiat Oncol Biol Phys.

[17] Im H-J, Bradshaw T, Solaiyappan M, Cho SY (2017). Current methods to define metabolic tumor volume in positron emission tomography: which one is better?. Nucl Med Mol Imaging.

[18] Hyun SH, Choi JY, Shim YM, Kim K, Lee SJ, Cho YS (2009). Prognostic value of metabolic tumor volume measured by 18F-Fluorodeoxyglucose positron emission tomography in patients with esophageal carcinoma. Ann Surg Oncol.

[19] Hyun SH, Ahn HK, Kim H, Ahn M-J, Park K, Ahn YC (2014). Volume-based assessment by (18) F-FDG PET/CT predicts survival in patients with stage III non-small-cell lung cancer. Eur J Nucl Med Mol Imaging.

[20] Chen HHW, Chiu N-T, Su W-C, Guo H-R, Lee B-F (2012). Prognostic value of whole-body total lesion glycolysis at pretreatment FDG PET/CT in non–small cell lung cancer. RY.

[21] Kim BS, Kim IJ, Kim S-J, Nam H-Y, Pak KJ, Kim K (2010). The prognostic value of the metabolic tumor volume in FIGO stage IA to IIB cervical cancer for tumor recurrence: measured by F-18 FDG PET/CT. Nucl Med Mol Imaging.

[22] Lim R, Eaton A, Lee NY, Setton J, Ohri N, Rao S (2012). 18F-FDG PET/CT metabolic tumor volume and Total lesion glycolysis predict outcome in oropharyngeal squamous cell carcinoma. J Nucl Med.

[23] Myerson RJ, Kong F, Birnbaum EH, Fleshman JW, Kodner IJ, Picus J (2001). Radiation therapy for epidermoid carcinoma of the anal canal, clinical and treatment factors associated with outcome. Radiother Oncol.

[24] Akbari RP, Paty PB, Guillem JG, Weiser MR, Temple LK, Minsky BD (2004). Oncologic outcomes of salvage surgery for epidermoid carcinoma of the anus initially managed with combined modality therapy. Dis Colon Rectum.

[25] Roohipour R, Patil S, Goodman KA, Minsky BD, Wong WD, Guillem JG (2008). Squamous-cell carcinoma of the anal canal: predictors of treatment outcome. Dis Colon Rectum.

[26] Mistrangelo M, Pelosi E, Bellò M, Ricardi U, Milanesi E, Cassoni P (2012). Role of positron emission tomography-computed tomography in the management of anal cancer. Int J Radiat Oncol Biol Phys.

[27] Cardenas ML, Spencer CR, Markovina S, DeWees TA, Mazur TR, Weiner AA (2017). Quantitative FDG-PET/CT predicts local recurrence and survival for squamous cell carcinoma of the anus. Adv Radiat Oncol.

[28] Wright CL, Binzel K, Zhang J, Knopp MV (2017). Advanced functional tumor imaging and precision nuclear medicine enabled by digital PET technologies. Contrast Media Mol Imaging.

